# A longitudinal two-year survey of the prevalence of trypanosomes in domestic cattle in Ghana by massively parallel sequencing of barcoded amplicons

**DOI:** 10.1371/journal.pntd.0010300

**Published:** 2022-04-20

**Authors:** Jennifer Afua Ofori, Soale Majeed Bakari, Saikou Bah, Michael Kojo Kolugu, George Kwame Aning, Gordon Akanzuwine Awandare, Mark Carrington, Theresa Manful Gwira

**Affiliations:** 1 West African Centre for Cell Biology of Infectious Pathogens, College of Basic and Applied Sciences, University of Ghana, Accra, Ghana; 2 Department of Biochemistry, Cell and Molecular Biology, College of Basic and Applied Sciences, University of Ghana, Accra, Ghana; 3 Department of Computer Science, College of Basic and Applied Sciences, University of Ghana, Accra, Ghana; 4 School of Veterinary Medicine, College of Basic and Applied Sciences, University of Ghana, Accra, Ghana; 5 Department of Biochemistry, University of Cambridge, Cambridge, United Kingdom; Fundaçao Oswaldo Cruz, BRAZIL

## Abstract

**Background:**

Animal African Trypanosomiasis (AAT) is one of the most economically important diseases affecting livestock productivity in sub-Saharan Africa. The disease is caused by a broad range of *Trypanosoma* spp., infecting both wild and domesticated animals through cyclical and mechanical transmission. This study aimed to characterize trypanosomes present in cattle at regular intervals over two years in an AAT endemic and a non-endemic region of Ghana.

**Methodology/Principal findings:**

Groups of cattle at Accra and Adidome were selected based on their geographical location, tsetse fly density, prevalence of trypanosomiasis and the breed of cattle available. Blood for DNA extraction was collected at approximately four to five-week intervals over a two-year period. Trypanosome DNA were detected by a sensitive nested PCR targeting the tubulin gene array and massively parallel sequencing of barcoded amplicons. Analysis of the data was a semi-quantitative estimation of infection levels using read counts obtained from the sequencing as a proxy for infection levels. Majority of the cattle were infected with multiple species most of the time [190/259 (73%) at Adidome and 191/324 (59%) at Accra], with *T*. *vivax* being the most abundant. The level of infection and in particular *T*. *vivax*, was higher in Adidome, the location with a high density of tsetse flies. The infection level varied over the time course, the timings of this variation were not consistent and in Adidome it appeared to be independent of prophylactic treatment for trypanosome infection. Effect of gender or breed on infection levels was insignificant.

**Conclusions/Significance:**

Most cattle were infected with low levels of several trypanosome species at both study sites, with *T*. *vivax* being the most abundant. The measurements of infection over time provided insight to the importance of the approach in identifying cattle that could suppress trypanosome infection over an extended time and may serve as reservoir.

## Author summary

Cattle are of economic importance in sub-Saharan Africa as they fulfil multiple roles, ranging from draught power, to providing manure, milk, and meat. However, Animal African Trypanosomiasis (AAT) diseases in cattle affect productivity and food security in most African countries. In Ghana, bovine trypanosomiasis has been detected in few cross-sectional studies by molecular methods. To get a better understanding of the disease, a longitudinal study showing natural trypanosome infection over the life of cattle will be applicable. To explore this issue, the study determined the nature of trypanosome infection in cattle in farm settings in Ghana over two years, a period similar to that used in beef production, by massively parallel amplicon sequencing. The study provided a description of the prevalence over two years and showed that the cattle were infected with multiply species most of the time and the level of infection varied but was low most of the time. The longitudinal study allowed the identification of one individual able to supress infection far more effectively than other members of the herd and this is crucial in implementing control measures in the infected area.

## Introduction

Animal African Trypanosomiasis (AAT) is a disease caused by extracellular protozoan pathogens of the genus *Trypanosoma* and is present in most or all sub-Saharan countries. Infection occurs in a wide range of domestic animals including cattle, sheep, goats, pigs and horses. The economic consequences of livestock infection disproportionally affect the rural, and often marginalised, communities that have benefited least from economic development and cannot afford to pay for treatment. The presence of AAT directly causes considerable losses in productivity of livestock and an estimated millions of animals and humans are at risk of the disease [1–5].

A range of trypanosome species have been detected in livestock. In Africa, there are predominantly Salivarian species, transmitted in saliva by biting flies, and include: *Trypanosoma brucei*, *T*. *congolense*, *T*. *evansi* (an immediate derivative of *T*. *brucei* restricted to blood transmission [6]) *T*. *godfreyi*, *T*. *simiae* and *T*. *vivax* [7–10]. In addition, cattle are often infected with *T*. *theileri*, a Stercorarian species transmitted through the faeces of biting flies, and phylogenetically distant from the other species [7–10]. *T*. *brucei*, *T*. *congolense* and *T*. *vivax* are found in most types of livestock and are present in a wild animal reservoir such as lion, baffalo and warthog [11]. These three species are the most economically important trypanosomes responsible for considerable production losses and livestock morbidity [4,9,12]. The two subspecies of *T*. *brucei*, *T*. *gambiense* and *T*. *rhodesiense*, have been shown to be zoonotic with both domestic and wild hosts, which impact greatly on control measures and the sustainability of zero cases in humans [13,14]. *T*. *simiae* and *T*. *godfreyi* are found mainly in domestic and wild pigs. *T*. *theileri* has a worldwide distribution and is usually non-pathogenic [8,15–17]. The most common trypanosome species detected in cattle in Ghana are *T*. *brucei*, *T*. *congolense*, *T*. *vivax*, *T*. *theileri* and *T*. *simiae* [18,19].

Whilst a number of studies have shown the distribution and prevalence of the trypanosome species that affect animals in Africa using different methodologies, molecular diagnostic techniques for rapid, sensitive and specific detection of trypanosomes such as polymerase chain reaction (PCR) can be used to detect both single and multiple infections [19–21]. Most methodologies for the identification of trypanosomes in both the animal host and the insect vector have made used of the amplification of the internal transcribed spacer sequence either in a conventional PCR or PCR coupled with sequencing [8,15,18,19,22] and these have been done using either small or large animal population in cross-sectional studies. In Ghana, only a few studies have used molecular methods to identify trypanosomes species and these were cross-sectional [18,19,23,24].

To our knowledge, there is no longitudinal study that shows natural trypanosomes infection over the life of an animal. This study characterized trypanosomes present in cattle at regular intervals over two years in an AAT endemic region and a non-endemic region of Ghana by massively parallel amplicon sequencing. The objectives were: (i) to determine the prevalence of infection; identify the infecting species; and (ii) to identify any internal (breed, age and gender) and external (tsetse fly density and treatment) factors that may affect infection. The findings provide a description of the prevalence over two years and show that the cattle were infected with more than one species most of the time, the level of infection fluctuated but was probably low most of the time, and *T*. *vivax* was the predominant species in cattle from the endemic area. The analysis also allowed the identification of one individual able to supress infection far more effectively than other members of the herd.

## Methods

### Ethics statement

Ethical approval for this study was obtained from the Council for Scientific and Industrial Research (CSIR) Institutional Animal Care and Use Committee (IACUC), Ghana (RPN 001/CSIR-IACUC/2013).

### Study area and animal population

The study was carried out at two different cattle trypanosomiasis endemicity areas in Ghana [18,19,25] to determine the influence of the environment on lifetime natural trypanosomes infections. First, a commercial cattle ranch at Adidome in the transitional forest vegetation zone (latitude 6° 04’ 25.97" North and longitude 0° 29’ 59.88" East), an area with abundant tsetse flies and high endemic AAT. Second, the University of Ghana Livestock and Poultry Research Farm in the Greater Accra region in the coastal savanna vegetation zone (latitude 5° 40’ 29.20" North and longitude 0° 06’ 14.95" West), an area with very few tsetse flies and low endemic AAT [25] (S1 Fig). Cattle used for the studies were selected as typical for those present on farms in the two respective locations. They were aged between 2 to 7 months at the beginning of the study and included both sexes. The breeds of cattle at the Accra study site were Sanga, Sanga Cross and West African Short Horn (WASH) whereas at Adidome study site all were Sanga. A total of 36 cattle was randomly selected, 20 in Accra and 16 in Adidome, from each study sites and ear-tagged for identification during follow up. S1 Table contains the information on the age, sex and breeds of cattle selected at the two study sites. Animals at Adidome were periodically treated for AAT with one or both of Isometamidium chloride (0.025g/300kg (w/w)) or Diminazene aceturate (1.05g/300 kg (w/w)), this was part of normal practice for cattle rearing on the farm because of the high prevalence of AAT and a previous history of AAT outbreak [19] (S1A Table). No treatment for AAT was administered to animals at Accra. Occasionally, it was not possible to collect samples from individual cattle and some cattle died or were sold during the course of the study (S1 Table). One limitation of the study is that we did not document the animals body score, however loss of weight was visually observed in a few of the animals.

### Sample collection and DNA isolation

Peripheral blood samples were collected from cattle at approximately four to five weeks interval from both study sites over 2 years giving a total of 18 time points. At each timepoint, a 5 ml blood sample was collected from the jugular vein into an S-monovette blood collection tube coated with Ethylenediaminetetraacetic acid (EDTA) (Sarstedt). Samples were placed on ice during transportation to the laboratory. DNA was extracted from ~5 ml whole blood samples using the QIAamp DNA Blood Maxi Kit (Qiagen), following the manufacturer’s protocol giving a final volume of 1 ml of DNA solution.

### Identification of trypanosome species using multiplexed nested PCR

Trypanosome DNA was detected by amplification of the region between the alpha and beta tubulin coding sequences using a nested PCR. The oligonucleotide primers were designed using available sequences from the Salivarian species *T*. *brucei*, *T*. *congolense* and *T*. *vivax* and the distantly related Stercorarian species *T*. *theileri* (S1 Text). For the first round PCR, 1 μl of cattle whole blood DNA was added to 29 μl of PCR reaction mixture. The reaction mixture contained 1X Mango Taq buffer with 2.5 mM MgCl_2_, 0.3 μM outer primers, 0.3 mM dNTPs, and 2.5 U Mango *Taq* polymerase (Bioline Reagents). The thermal cycling condition were 94°C for 5 minutes, followed by 30 cycles of 94°C for 45 seconds, 61°C for 45 seconds, 72°C for 45 seconds plus 3 seconds per cycle, and a final extension of 72°C for 5 minutes then transfer to 4°C. The second round of PCR used a reaction mix of 50 μl containing 1 μl of the first PCR and 49 μl of the reaction mix above except that the outer primers were replaced with inner primers. When preparing PCR products for multiplex sequencing, the forward inner oligo was barcoded to identify the individual cow and the reverse inner oligo for the time point (S2 Table). Genomic DNA from *T*. *b*. *brucei* and PCR water was used as positive and negative controls respectively.

### Next generation sequencing library preparation and targeted amplicon sequencing

Amplicons generated from the multiplex tagged *Trypanosoma* tubulin nested PCR were pooled for Next Generation Sequencing (NGS). The sequencing library was prepared and sequenced in the Sequencing Facility at the Department of Biochemistry, University of Cambridge. Whole PCR amplicons were sequenced using paired end reads after ligation of adaptors to the ends. Each sample was uniquely indexed with Illumina Nextera XT indices, and libraries for individual samples pooled and prepared using the Nextera XT DNA sample preparation kit according to the manufacturer’s protocol. The library was sequenced on an Illumina MiSeq using a 600-cycle kit V2 kit (Illumina, San Diego, USA) according to the manufacturer’s protocol producing 300 base paired-end reads.

### Quality control and mapping of sequence data

An in-house python script was used to de-multiplex the reads into individual cows and their respective time points based on their unique tags. FastQC was used for read quality control [26]. Paired-end reads were then mapped to different trypanosome reference sequences using Bowtie2 and the default settings [27]. The quality of the sequence alignment mapping (SAM) files was assessed to remove those with low mapping quality and total mapped read counts were then obtained from the SAM files using SAMtools [28].

### Determination of trypanosomes infection

Relative abundance of infecting trypanosomes over the sampling period was calculated based on the number of trypanosomes derived read counts per animal per timepoint. Since the maximum number of reads that were allocated to all negative controls were less than 1000, an infection per specie was defined as read counts greater than 1000. Statistical analysis was done using the Mann Whitney U test (Wilcoxon rank-sum) for the various groups. Differences between groups were considered statistically significant at p < 0.05.

## Results

### Molecular genotyping of trypanosomes in cattle

Groups of cattle were chosen from two locations, a cattle ranch in Adidome with a high incidence of tsetse flies, and the University of Ghana Research Farm in Greater Accra with a low density. Groups of cattle were then followed over two years, a period similar to the normal cycle for beef production in Ghana. Over the course of these two years, the incidence of definitive symptoms of acute animal African trypanosomiasis was rare and only one of the cattle in the study at Adidome (Ad41) showed possible acute symptoms of AAT but this was not confirmed. The approach was to take blood from cattle, purify DNA and identify trypanosome DNA using nested PCR. The majority of previous studies have identified different trypanosome species using an amplicon based on variation in the length of an internal transcribed spacer (ITS) flanking the 5.8S rRNA gene (for example [22]). Here, an alternative nested PCR based on the tubulin locus was used. Trypanosome genomes contain an array of alternating α- and β-tubulin genes and, for example in one *T*. *brucei* isolate there are 19 copies per haploid genome [29], similar to the copy number for rRNA genes. Trypanosome species were detected and distinguished by amplification of part of the tubulin locus from close to the 3’ end of the α-tubulin open reading frame (ORF) to the 5’ end of the β-tubulin ORF (S1 Text). This genomic region was chosen as in preliminary experiments the PCR was more sensitive than the ITS-based assay when used with DNA purified from whole cattle blood.

Trypanosome species were initially identified based on the length of PCR amplicon and subsequently by next generation sequencing of pooled amplicons with barcoded primers indicating the identity of individual cattle and the time point. A total of 17,162,252 reads mapped to the various tubulin sequences after quality control and the removal of reads with incomplete barcodes. The number of trypanosome derived amplicon read counts per animal per timepoint was determined over the time course and used as a proxy for relative abundance (S3A Table). This makes some assumptions but is the best available method given the low levels of blood parasitaemia in most cattle. One consideration in the interpretation of the sequencing data is what a very low number of read counts indicates. Reads can be falsely attributed to a particular cow/timepoint due to a low frequency of errors during oligonucleotide synthesis, PCR errors that affect the barcode sequence, and sequencing errors in the barcodes and the large number of reads obtained means that such errors appear. We took advantage of the small number of absent samples (23 from 594, S1 Table) and allowed the allocation of reads to these by the software that deconvoluted the barcoding. The total number of reads for all trypanosome species allocated to absent samples was less 3298 (0.02%) indicating a very low frequency of misallocation of reads. For absent cattle-time points there were less than 100 reads for all Accra samples and less than 500 for Adidome samples except one (854 for cow Ad49 timepoint 9). This gave us confidence that any read count of more than 1000 for any individual species represented an infection but does not exclude lower counts resulting from an infection.

**Fig 1:**
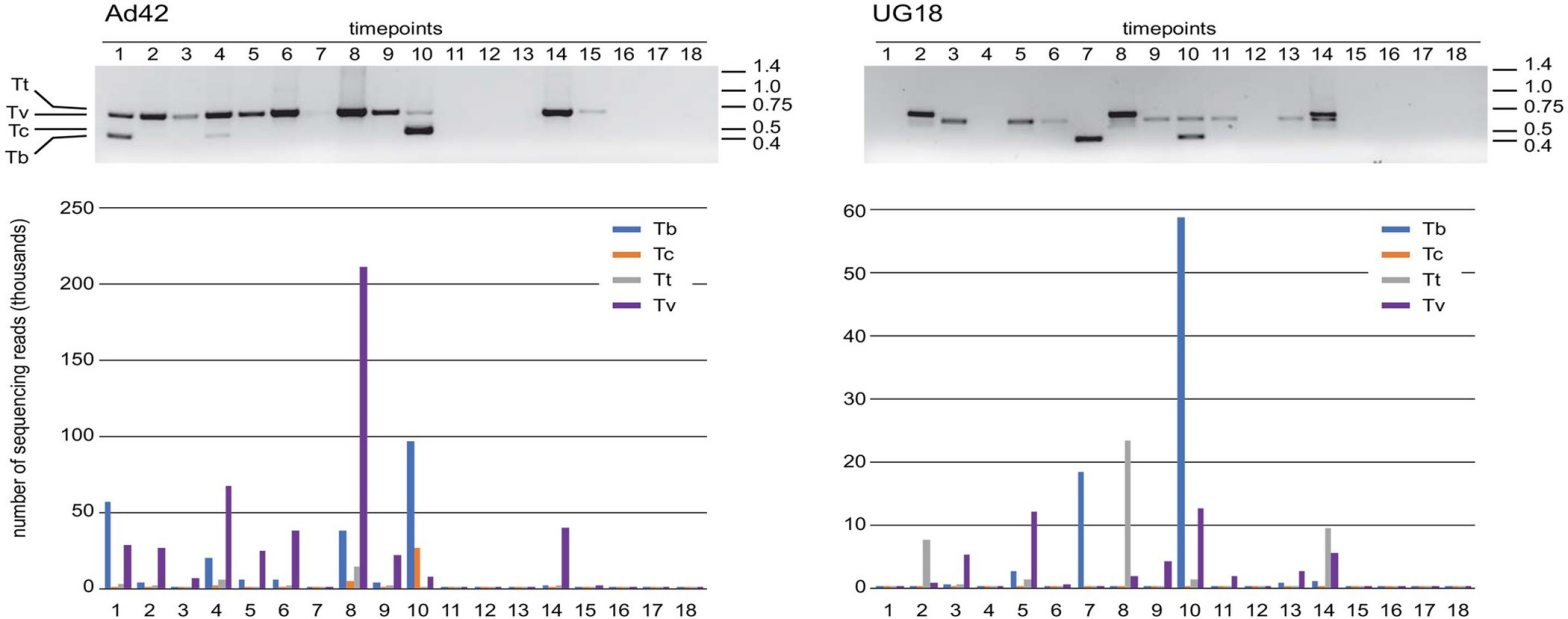
Detection of trypanosome species in cattle over 18 time points spanning two years. Two individuals are shown: Ad42 from Adidome and UG18 from Accra. Comparison of analysis by agarose gel electrophoresis (top) and read count after massively parallel amplicon sequencing (bottom). For the agarose gels, DNA was visualised using ethidium bromide and the image is shown as a negative. The species are indicated (Tt, *T*. *theileri*, Tv, *T*. *vivax*; Tc, *T*. *congolense*; Tb, *T*. *brucei*) and DNA size standards (kbp) are shown to the right of each gel. The read counts are shown for each species at each time point, note that the y-axis scales are different for each cow.

When the reads were allocated to individual species, four were routinely detected, *T*. *vivax*, *T*. *brucei*, *T*. *congolense* and *T*. *theileri*. The presence of other species cannot be ruled out but there was not a large number of unallocated reads. To rule out the zoonotic subspecies of *T*. *brucei*, PCR was done using *T*. *gambiense* specific primers on a few selected positive samples which came out negative. Fig 1 shows a comparison of amplicon products analysed by agarose gel electrophoresis and read counts over the entire time course for two cattle. There is a clear concordance between the bands visible on the gel and the relative abundance determined after sequencing. The amplicon sequencing was more sensitive than detection after gel electrophoresis and enabled detection and estimates of abundance of trypanosomes when no PCR product was visible after gel electrophoresis.

### Infections in herds

**Fig 2:**
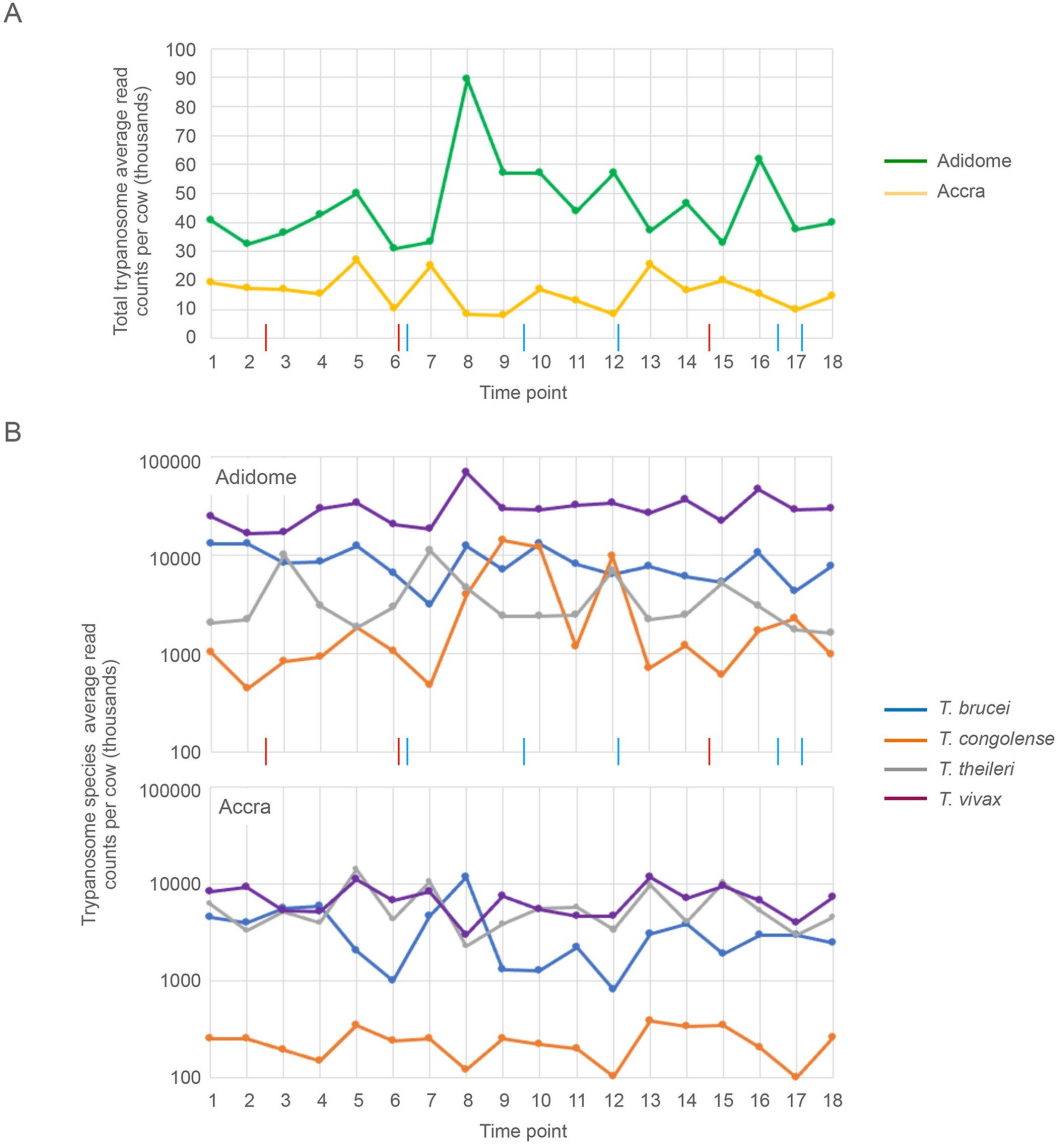
Infections in herds. (A) The average infection at each location over two years estimated by taking the average of the total trypanosome read counts for each cow at each time point at Adidome (green) and Accra (yellow). The timing of drug treatments at Adidome is indicated on the x-axis: blue, diminazene; red, isometamidium. (B) The incidence of four trypanosome species in the two herds over the time course expressed as the average of the species-specific read counts per cow at each time point. The colour code for each species is indicated and the y-axis is a logarithmic scale. The timing of drug treatments at Adidome is indicated on the x-axis: blue, diminazene; red, isometamidium.

The estimates of trypanosome infection were used to investigate any differences between the cattle at the two sites and other factors influencing the infection in the group as a whole before going on to infections in individual cattle. The two sites were compared by calculating a herd average infection by taking the average of the total read counts for each cow at each time point as a measure of trypanosome abundance (Fig 2A, and S3B and S3C Table). The infection in cattle at Adidome was greater at all 18 time points (p = 2.2e-10), this might be expected in an area of endemic AAT. There was a peak of infection in Adidome at time point 8 when the average read counts per cow increased threefold compared to the previous month but this did not correspond to any obvious external change. This comparison was repeated but using the average read counts for each trypanosome species at each time point (Fig 2B, and S3D and S3E Tables). This showed that there was a hierarchy of species present in the cattle at Adidome, with *T*. *vivax* being the most abundant followed by *T*. *brucei* and *T*. *theileri*. *T*. *congolense* was normally the least abundant but there were some time points where it exceeded *T*. *brucei*. In Accra, *T*. *vivax*, *T*. *brucei* and *T*. *theileri* were present at similar levels and *T*. *congolense* less abundant. The overall trypanosome prevalence was relatively constant over the two years and there was no obvious effect of age on trypanosome burden up to the age of 30 months.

**Fig 3:**
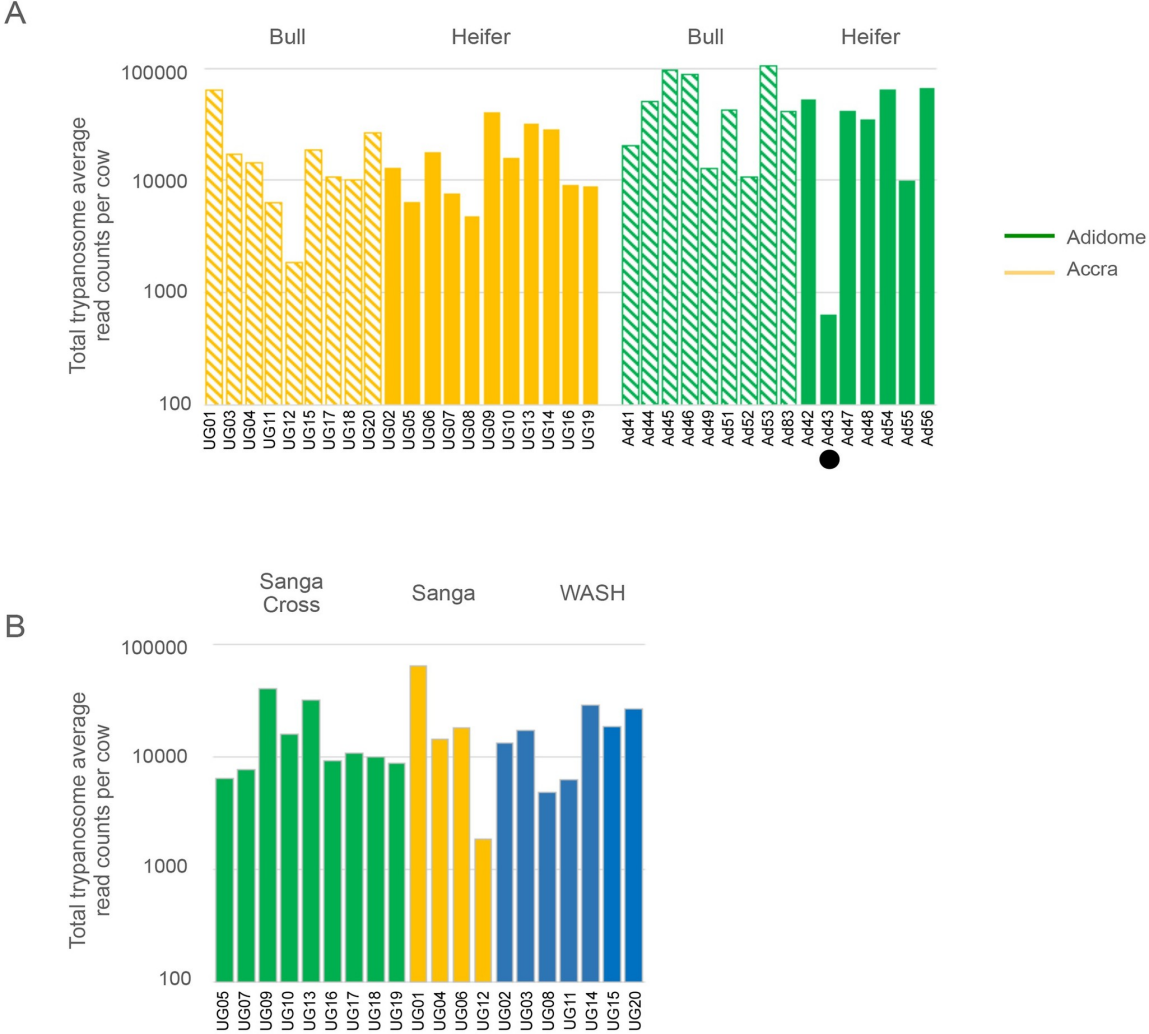
Infections by gender and breed. (A) Average infection over the time course estimated by taking the average read count in each animal and then separated by gender: bulls, hatched; heifers, solid, and by location: Adidome, green and Accra, orange. The colour code for each location is indicated and the y-axis is a logarithmic scale. Cow Ad43 is indicated by the black circle. (B) Average infection over the time course in Accra estimated by taking the average read count in each animal and then separated by cattle breed: green, Sanga Cross; yellow, Sanga; blue, West African Short Horn (WASH). The y-axis is a logarithmic scale.

To determine whether there was any obvious effect of gender or breed on levels of infection, the average read count per cow per time point was calculated and used as a measure of lifetime burden. When bulls and heifers were compared at both sites, there was no differences between them (Fig 3A, and S3F Table; Accra, p = 0.07; Adidome, p = 0.47 with Ad43 and p = 0.78 without Ad43). However, at both sites, bulls had a slightly higher burden but the difference probably does not indicate a difference in susceptibility. All the cattle at Adidome were Sanga, whereas at Accra the group contained West African Short Horn (WASH), Sanga and Sanga Cross. In terms of trypanotolerence, these are ranked WASH as most tolerant followed by Sanga and then Sanga Cross [30–32]. We found no significant difference in read counts between the three cattle types at Accra (Sanga v Sanga Cross, p = 0.60; Sanga v WASH, p = 1.07; WASH v Sanga Cross, p = 0.84) but noting that this is an area with low tsetse fly densities (Fig 3B and S3G Table).

### Infections in individual cattle

**Fig 4:**
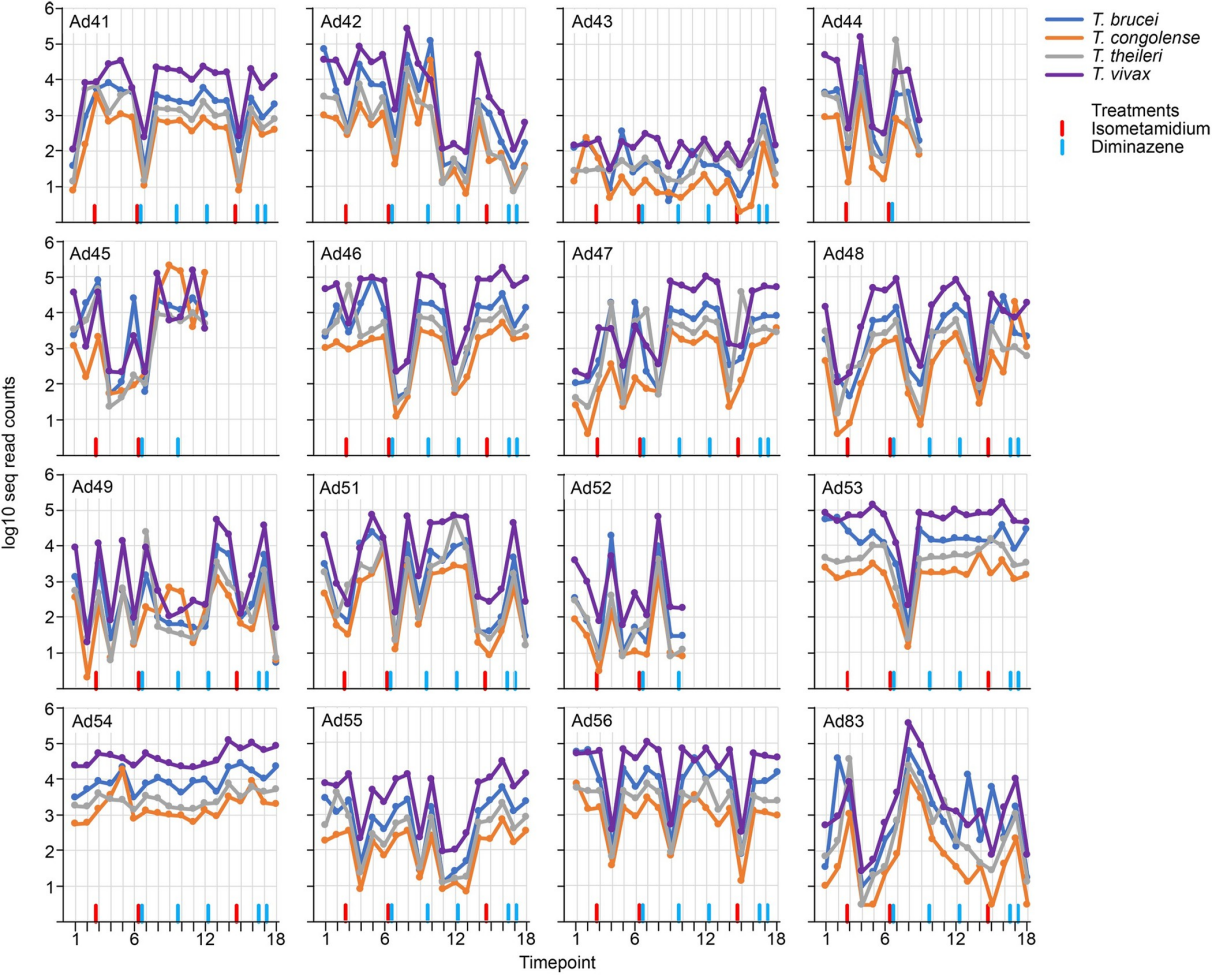
Infections of individual cattle with the different species of trypanosome over two years in Adidome. The colour code for each species is indicated: blue, *T*. *brucei*; orange, *T*. *congolense*; grey, *T*. *theileri*; purple, *T*. *vivax*. The timing of drug treatments at Adidome is indicated on the x-axis: blue, diminazene; red, isometamidium. The y-axis is a logarithmic scale.

**Fig 5:**
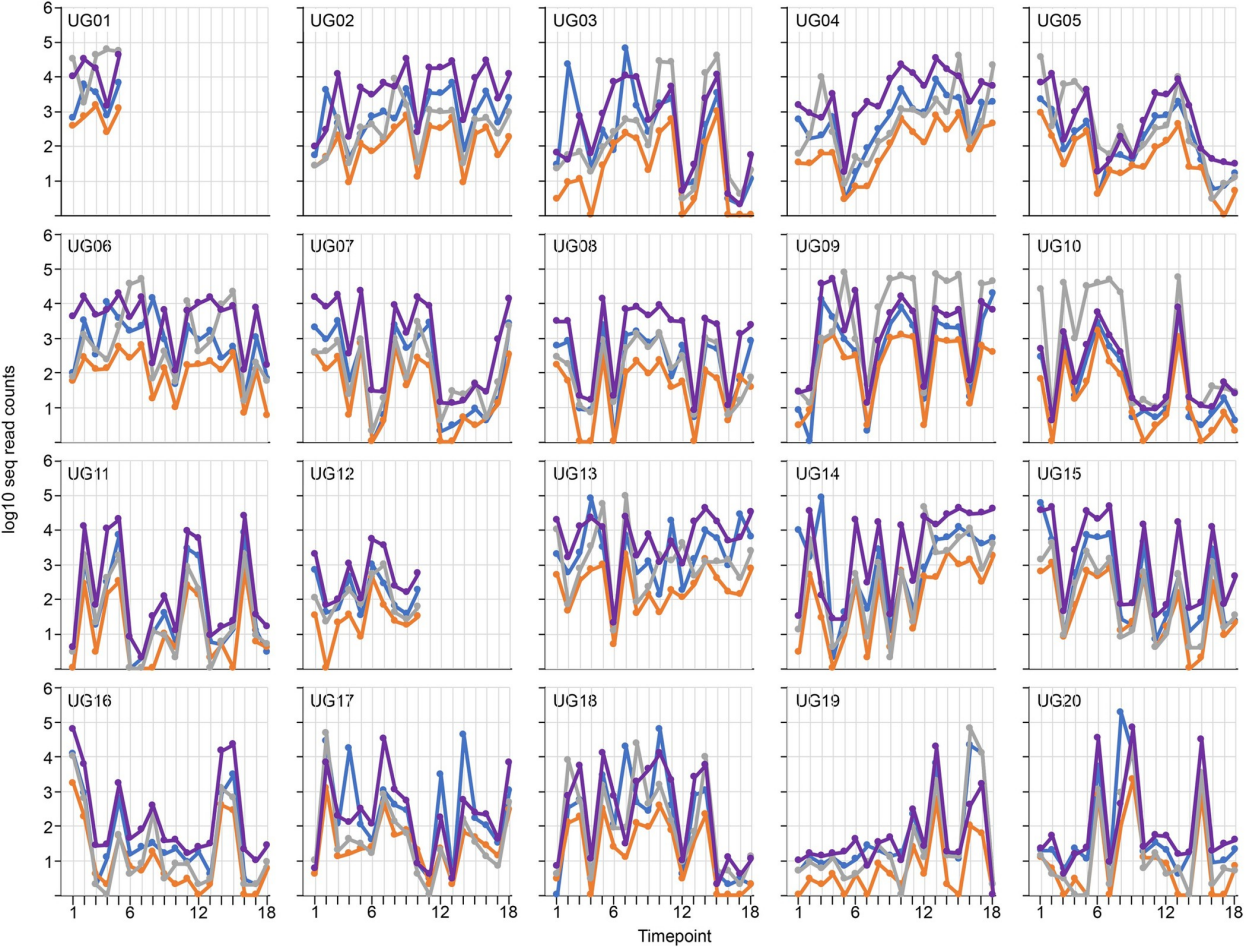
Infections of individual cattle with the different species of trypanosome over two years in Accra. The colour code for each species is indicated: blue, *T*. *brucei*; orange, *T*. *congolense*; grey, *T*. *theileri*; purple, *T*. *vivax*. The y-axis is a logarithmic scale.

The behaviour of the four trypanosome species in individual cattle was analysed at Adidome (Fig 4 and S3H Table) and Accra (Fig 5 and S3I Table) over the time course. Most of the cattle in Adidome had trypanosome infection most of the time throughout the year. After applying the 1000 read counts cut off to account for uncertainty of the significance of low reads, infection was detected in 190/259 (73%) samples and it is likely that the real figure is higher. The cattle were usually infected with most or all of the detectable species and the most abundant was usually but not always *T*. *vivax*, occasionally *T*. *brucei* or *T*. *congolense* was more abundant at some time points, for example in cows Ad42 and Ad45 (Fig 4). The infections fluctuated with peaks and troughs with often more than 1000-fold differences in read count numbers, for example cow Ad46 (Fig 4). The fluctuations in read counts were usually followed by all four species, for example the reduction and increase seen over time points 6 to 10 in cow Ad53 (Fig 4). However, this was not always the case and sometimes one species increased and one decreased between two time points, for example timepoints 12 to 17 in cow Ad83 (Fig 4).

The cattle at Adidome were given prophylactic treatment for trypanosome infections at seven separate occasions over the two years, the times of these treatments are in S1A Table and are shown in Figs 2 and 4. There was no evidence that the treatments had an immediate effect on trypanosome burden in the group of cattle as a whole (Fig 2), but some treatments may have been beneficial for individual cattle as they preceded a dramatic decrease in trypanosome infection, for example treatment between timepoints 6 and 7 for cows Ad41 and Ad46 (Fig 4). However, no consistent picture emerges of the effect of treatment on trypanosome burden but this is in a context of a herd in which acute AAT was extremely rare, so prophylaxis was successful. Moreover, the level of infection in cow AD43 was lower than all other cattle at Adidome, over the 18 timepoints and the average number of read counts was 15-fold lower than the next most lightly infected cow (Fig 3A). There was only one time point when Ad43 was found with a definite infection (>1000 read counts) (Fig 4). It appears that cow Ad43 was able to either avoid infection or more likely supress trypanosome infection very effectively when compared to other cattle in the same herd.

The cattle at Accra showed a similar pattern of infection to those in Adidome with peaks and troughs but had a lower overall infection level (Figs 5 and 2A) and frequency of infection. Using >1000 read counts as a cut off, 191/324 (59%) samples were positive for trypanosome infection and again the real figure is likely to be higher. As in Adidome, all trypanosome species usually, but not always, followed the same pattern of peaks and troughs, for example cows UG11 and UG15 (Fig 5). Although *T*. *vivax* was the most abundant species in most cattle and at most timepoints, both *T*. *brucei* and *T*. *theileri* were present at similar levels whereas *T*. *congolense* was the least abundant. (Figs 2B and 5) and this was reflected by many time points in individual cattle in which *T*. *brucei* or *T*. *theileri* was the most abundant species present, for example cows UG17 and UG10 (Fig 5).

## Discussion

This longitudinal study was conducted to provide information on the trypanosome species infecting cattle in a low and high tsetse fly endemic regions in Ghana over two years, a period similar to that used in beef production. The approach was to detect circulating trypanosome DNA by a sensitive nested PCR and massively parallel sequencing of barcoded amplicons. This approach has been successfully used before for the identification of trypanosomes present in tsetse flies [22]. Here, an amplicon derived from the tubulin gene array was used in contrast to one derived from a ribosomal RNA gene spacer in previous work [22]. The output of the analysis was a semi-quantitative estimation of infection levels using read counts obtained from the sequencing as a proxy for infection levels.

The findings of the work were: (i) the majority of the cattle were infected with multiple species most of the time with *T*. *vivax* being the most abundant followed by *T*. *brucei*, *T*, *theileri* and then *T*. *congolense* being the least abundant in most cows at most time points. (ii) The level of infection and in particular *T*. *vivax*, was higher in Adidome the location with a high density of tsetse flies. (iii) The infection level varied over the time course with troughs and peaks, the timings of this variation were not consistent between cattle in the study group and in Adidome appeared to be independent of prophylactic treatment for trypanosome infection. (iv) Infection levels neither increased nor decreased over the two years. (v) There was no obvious effect of gender or breed on infection levels although the numbers of cattle in the study was not large enough for a rigorous analysis.

The finding that there is a high frequency of infection and that *T*. *vivax* is the predominant infecting species in cattle in Ghana is consistent with previous work [18,19,25]. Similarly high rates of infection have been detected in cattle in Nigeria [33,34] with *T*. *vivax* as the most abundant species. One difference between the observations here and other surveys is the low abundance of *T*. *congolense* which was less abundant than both *T*. *brucei* and *T*. *theileri*.

The presence and abundance of *T*. *theileri* is informative. It is a stercorarian trypanosome distantly related to the species that cause AAT, it only infects bovids and is characterized by a low but persistent parasitemia [35,36] only causing disease downstream of high parasitaemia in extremely rare instances [37]. Transmission of *T*. *theileri* is not dependent on tsetse flies but occurs through biting flies and possibly through ingestion of infected fly faeces by cattle. In Accra, the read counts for *T*. *theileri* were similar to those from *T*. *brucei* and *T*. *vivax* and greater than those for *T*. *congolense*. This may indicate that the level of infection with all trypanosome species was low and possibly transmitted by biting flies or some other form of direct transmission. This is consistent with the low levels of *T*. *congolense* as it is mainly transmitted cyclically by the tsetse fly and to a lesser extent by mechanical means [38]. Thus, the lower frequency in Accra may be attributed to the known low tsetse fly challenge at this location.

In Adidome, the read counts for *T*. *brucei* and *T*. *congolense* were similar to those for *T*. *theileri*, again suggesting low levels of infection. In contrast, *T*. *vivax* was consistently present at a higher parasitaemia, consistent with transmission by tsetse flies. However, the rarity of acute AAT suggests that the regular prophylactic treatment was successful in suppressing high levels of infection. The continual low levels of infection suggest a possible drug resistance and/or continual re-infection and there have been reports in other African countries of the existence of trypanocidal drug resistance (diminazene aceturate and isometamidium chloride) among trypanosomes in cattle [39,40]. The high frequency of low levels of infection and rarity of acute symptoms of AAT are also consistent with trypanotolerance, the cattle included both trypanotolerant (WASH) and cross-breeds with trypanotolerant breeds (Sanga and Sanga Cross). Trypanotolerance is likely playing a role in the animals through controlling the levels of parasitemia [41]. In both locations cattle were infected from the first time point at 3 to 6 months old. This indicates high rates of infection and/or vertical transmission.

The value of longitudinal studies is exemplified by the analysis of cow Ad43 which had exceptional low parasitaemia over the time course. The total measure of infection was more than tenfold lower than any other cow at Adidome. In a single time point survey, a number of the other cows would have had a low parasitaemia but would not have identified Ad43 as particularly able to suppress infection. An expansion of the investigation described here would identify a sufficient number of cows like Ad43 to both select for breeding but also to provide insight in to any genetic basis for the suppression of infection.

The approach described here has limitations for quantitation of infection, the level of amplicon will be affected by the copy number of the target sequence in the genome and this may vary between species making direct comparisons difficult. The substrate for the amplification is blood DNA and the concentration of DNA will be affected by the location of the trypanosomes in the host, blood or tissues. Also, limitation associated to sampling include the unequal number of breeds at the two sampling sites, loss of some of the animals over the study period and low parasitemia that may affect detection. Thus, the measurements here are presented as estimates but the values for any one species are almost certainly a good representation of fluctuations in infection.

Overall, the work described here has shown that, at the two locations in Ghana in the study, most cattle were infected with low levels of several trypanosome species. In an area with high tsetse fly density there was greater trypanosome infection, in particular *T*. *vivax* and to a lesser extent *T*. *congolense* with an increase in average *T*. *vivax* parasitemia. Gender and breed of cattle had no obvious effect on infection. The measurements of infection over a time course highlighted the importance of such an approach in identifying cattle that could suppress trypanosome infection over an extended time period and thus may serve as reservoir.

## Supporting information

S1 Text Oligonucleotide sequences for the nested tubulin intergenic PCR. (A) Shows the sequences. (B) Shows the location of the oligos in the relevant parts of the genomes from *T*. *brucei*, *T*. *congolense*, *T*. *vivax* and *T*. *theileri*.
(DOCX)

S1 Fig Sampling locations. Map was drawn using the ArcGiS version 10. Source map: https://www.arcgis.com/apps/mapviewer/index.html?webmap=a52ab98763904006aa382d90e906fdd5.
(TIF)

S1 Table Details of age, sex and breed of cattle used in the study. The cattle at each site are listed in the separate tables. (S1A) The time points for collection of blood and various drug treatments for the Adidome site. AAT treatments in red and light blue, for other infections in grey. (S1B) The time points for collection of blood for analysis are shown for the Accra site.
(XLSX)

S2 Table Barcoded oligonucleotide sequences used for second amplification prior to sequencing. (XLSX)

S3 Table Number and analysis of read counts for cattle over the two years of the study. (S3A) Total trypanosome read counts allocated to individual cattle and time points. (S3B) Calculation of herd averages for total trypanosome reads. (S3C) Variation in total trypanosomes over the time course at each location. (S3D) Calculation of herd averages for each trypanosome species. (S3E) Herd average read counts for each trypanosome species at each timepoint. (S3F) Average read counts per cow over 18 timepoints separated by gender. (S3G) Average read counts in Accra over 18 timepoints separated by breed. (S3H) Read counts for trypanosomes species in individual cattle at Adidome. (S3I) Read counts for trypanosome species in individual cattle in Accra. (S3J) Average read counts per cow over the time course.
(XLSX)

## References

[1] Dagnachew S, Bezie M. Review on *Trypanosoma vivax*. African Journal of Basic & Applied Sciences. 2015;7(1):41–64.

[2] Dagnachew S, Bezie M, Terefe G, Abebe G, Barry JD, Goddeeris BM. Comparative clinico-haematological analysis in young Zebu cattle experimentally infected with *Trypanosoma vivax* isolates from tsetse infested and non-tsetse infested areas of Northwest Ethiopia. Acta Vet Scand. 2015;57:24. doi: 10.1186/s13028-015-0114-2 25986858 PMC4450476

[3] Yaro M, Munyard KA, Stear MJ, Groth DM. Combatting African Animal Trypanosomiasis (AAT) in livestock: The potential role of trypanotolerance. Vet Parasitol. 2016;225:43–52. doi: 10.1016/j.vetpar.2016.05.003 27369574

[4] Giordani F, Morrison LJ, Rowan TG, HP DEK, Barrett MP. The animal trypanosomiases and their chemotherapy: a review. Parasitology. 2016;143(14):1862–89. doi: 10.1017/S0031182016001268 27719692 PMC5142301

[5] Shaw AP, Wint GR, Cecchi G, Torr SJ, Mattioli RC, Robinson TP. Mapping the benefit-cost ratios of interventions against bovine trypanosomosis in Eastern Africa. Prev Vet Med. 2015;122(4):406–16. doi: 10.1016/j.prevetmed.2015.06.013 26166771

[6] Lai DH, Hashimi H, Lun ZR, Ayala FJ, Lukes J. Adaptations of *Trypanosoma brucei* to gradual loss of kinetoplast DNA: *Trypanosoma equiperdum* and *Trypanosoma evansi* are petite mutants of *T*. *brucei*. Proc Natl Acad Sci U S A. 2008;105(6):1999–2004. doi: 10.1073/pnas.0711799105 18245376 PMC2538871

[7] Ahmed HA, Picozzi K, Welburn SC, MacLeod ET. A comparative evaluation of PCR- based methods for species- specific determination of African animal trypanosomes in Ugandan cattle. Parasit Vectors. 2013;6(1):316. doi: 10.1186/1756-3305-6-316 24499678 PMC4029050

[8] Cox A, Tilley A, McOdimba F, Fyfe J, Eisler M, Hide G, et al. A PCR based assay for detection and differentiation of African trypanosome species in blood. Exp Parasitol. 2005;111(1):24–9. doi: 10.1016/j.exppara.2005.03.014 16054487

[9] Morrison LJ, Vezza L, Rowan T, Hope JC. Animal African Trypanosomiasis: Time to increase focus on clinically relevant parasite and host species. Trends Parasitol. 2016;32(8):599–607. doi: 10.1016/j.pt.2016.04.012 27167665

[10] Van den Bossche P. Some general aspects of the distribution and epidemiology of bovine trypanosomosis in southern Africa. Int J Parasitol. 2001;31(5–6):592–8. doi: 10.1016/s0020-7519(01)00146-1 11334947

[11] Anderson NE, Mubanga J, Fevre EM, Picozzi K, Eisler MC, Thomas R, et al. Characterisation of the wildlife reservoir community for human and animal trypanosomiasis in the Luangwa Valley, Zambia. PLoS Negl Trop Dis. 2011;5(6):e1211. doi: 10.1371/journal.pntd.0001211 21713019 PMC3119639

[12] Geerts S, Holmes PH, Eisler MC, Diall O. African bovine trypanosomiasis: the problem of drug resistance. Trends Parasitol. 2001;17(1):25–8. doi: 10.1016/s1471-4922(00)01827-4 11137737

[13] Simarro PP, Cecchi G, Franco JR, Paone M, Diarra A, Ruiz-Postigo JA, et al. Estimating and mapping the population at risk of sleeping sickness. PLoS Negl Trop Dis. 2012;6(10):e1859. doi: 10.1371/journal.pntd.0001859 23145192 PMC3493382

[14] Onyango RJ, Van Hoeve K, De Raadt P. The epidemiology of *Trypanosoma rhodesiense* sleeping sickness in Alego location, Central Nyanza, Kenya. I. Evidence that cattle may act as reservoir hosts of trypanosomes infective to man. Trans R Soc Trop Med Hyg. 1966;60(2):175–82. doi: 10.1016/0035-9203(66)90024-1 5950928

[15] Cox AP, Tosas O, Tilley A, Picozzi K, Coleman P, Hide G, et al. Constraints to estimating the prevalence of trypanosome infections in East African zebu cattle. Parasit Vectors. 2010;3:82. doi: 10.1186/1756-3305-3-82 20815940 PMC2944308

[16] Magona JW, Walubengo J, Odimim JJ. Differences in susceptibility to trypanosome infection between Nkedi Zebu and Ankole cattle, under field conditions in Uganda. Ann Trop Med Parasitol. 2004;98(8):785–92. doi: 10.1179/000349804225021532 15667711

[17] Mbewe NJ, Namangala B, Sitali L, Vorster I, Michelo C. Prevalence of pathogenic trypanosomes in anaemic cattle from trypanosomosis challenged areas of Itezhi-tezhi district in central Zambia. Parasit Vectors. 2015;8:638. doi: 10.1186/s13071-015-1260-0 26669306 PMC4681019

[18] Bakari SM, Ofori JA, Kusi KA, Aning GK, Awandare GA, Carrington M, et al. Serum biochemical parameters and cytokine profiles associated with natural African trypanosome infections in cattle. Parasit Vectors. 2017;10(1):312. doi: 10.1186/s13071-017-2255-9 28655350 PMC5488482

[19] Nakayima J, Nakao R, Alhassan A, Mahama C, Afakye K, Sugimoto C. Molecular epidemiological studies on animal trypanosomiases in Ghana. Parasit Vectors. 2012;5:217. doi: 10.1186/1756-3305-5-217 23025330 PMC3480844

[20] Takeet MI, Fagbemi BO, De Donato M, Yakubu A, Rodulfo HE, Peters SO, et al. Molecular survey of pathogenic trypanosomes in naturally infected Nigerian cattle. Res Vet Sci. 2013;94(3):555–61. doi: 10.1016/j.rvsc.2012.10.018 23245680

[21] Tasew S, Duguma R. Cattle anaemia and trypanosomiasis in western Oromia State, Ethiopia. Revue Méd Vét. 2012;163(12):581–8.

[22] Gaithuma AK, Yamagishi J, Martinelli A, Hayashida K, Kawai N, Marsela M, et al. A single test approach for accurate and sensitive detection and taxonomic characterization of trypanosomes by comprehensive analysis of internal transcribed spacer 1 amplicons. PLoS Negl Trop Dis. 2019;13(2):e0006842. doi: 10.1371/journal.pntd.0006842 30802245 PMC6414030

[23] Mahama CI, Desquesnes M, Dia ML, Losson B, De Deken R, Geerts S. A cross-sectional epidemiological survey of bovine trypanosomosis and its vectors in the Savelugu and West Mamprusi districts of northern Ghana. Vet Parasitol. 2004;122(1):1–13. doi: 10.1016/j.vetpar.2004.03.009 15158552

[24] Mahama CI, Desquesnes M, Dia ML, Losson B, De Deken R, Speybroeck N, et al. A longitudinal epidemiological survey of bovine trypanosomosis and its vectors in the White Volta river basin of Northern Ghana. Vet Parasitol. 2005;128(3–4):201–8. doi: 10.1016/j.vetpar.2004.12.007 15740857

[25] Mahama CI, Mohammed HA, Abavana M, Sidibe I, Kone A, Geerts S. Tsetse and trypanosomoses in Ghana in the twentieth century: a Review. Revue Élev MÉd vÉt Pays trop. 2003;56 (1–2):27–32.

[26] Andrews S. FastQC: a quality control tool for high throughput sequence data. http://www.bioinformatics.babraham.ac.uk/projects/fastqc. 2010.

[27] Li H, Durbin R. Fast and accurate short read alignment with Burrows-Wheeler transform. Bioinformatics. 2009;25(14):1754–60. doi: 10.1093/bioinformatics/btp324 19451168 PMC2705234

[28] Li H, Handsaker B, Wysoker A, Fennell T, Ruan J, Homer N, et al. The Sequence Alignment/Map format and SAMtools. Bioinformatics. 2009;25(16):2078–9. doi: 10.1093/bioinformatics/btp352 19505943 PMC2723002

[29] Ersfeld K, Asbeck K, Gull K. Direct visualisation of individual gene organisation in *Trypanosoma brucei* by high-resolution in situ hybridisation. Chromosoma. 1998;107(4):237–40. doi: 10.1007/s004120050302 9745048

[30] Makina SO, Whitacre LK, Decker JE, Taylor JF, MacNeil MD, Scholtz MM, et al. Insight into the genetic composition of South African Sanga cattle using SNP data from cattle breeds worldwide. Genet Sel Evol. 2016;48(1):88. doi: 10.1186/s12711-016-0266-1 27846793 PMC5111355

[31] Pienaar L, Grobler JP, Scholtz MM, Swart H, Ehlers K, Marx M, et al. Genetic diversity of Afrikaner cattle in southern Africa. Trop Anim Health Prod. 2018;50(2):399–404. doi: 10.1007/s11250-017-1447-9 29043474

[32] Taye M, Kim J, Yoon SH, Lee W, Hanotte O, Dessie T, et al. Whole genome scan reveals the genetic signature of African Ankole cattle breed and potential for higher quality beef. BMC Genet. 2017;18(1):11. doi: 10.1186/s12863-016-0467-1 28183280 PMC5301378

[33] Majekodunmi AO, Fajinmi A, Dongkum C, Picozzi K, Thrusfield MV, Welburn SC. A longitudinal survey of African animal trypanosomiasis in domestic cattle on the Jos Plateau, Nigeria: prevalence, distribution and risk factors. Parasit Vectors. 2013;6(1):239. doi: 10.1186/1756-3305-6-239 23958205 PMC3765779

[34] Odeniran PO, Macleod ET, Ademola IO, Welburn SC. Molecular identification of bovine trypanosomes in relation to cattle sources in southwest Nigeria. Parasitol Int. 2019;68(1):1–8. doi: 10.1016/j.parint.2018.09.005 30243980

[35] Kelly S, Ivens A, Mott GA, O’Neill E, Emms D, Macleod O, et al. An Alternative strategy for trypanosome survival in the mammalian bloodstream revealed through genome and transcriptome analysis of the ubiquitous bovine parasite *Trypanosoma* (Megatrypanum) *theileri*. Genome Biol Evol. 2017;9(8):2093–109. doi: 10.1093/gbe/evx152 28903536 PMC5737535

[36] Mott GA, Wilson R, Fernando A, Robinson A, Macgregor P, Kennedy D, et al. Targeting cattle-borne zoonoses and cattle pathogens using a novel trypanosomatid-based delivery system. PLoS Pathog. 2011;7(10). doi: 10.1371/journal.ppat.1002340 22046137 PMC3203185

[37] Doherty ML, Windle H, Voorheis HP, Larkin H, Casey M, Clery D, et al. Clinical disease associated with *Trypanosoma theileri* infection in a calf in Ireland. Vet Rec. 1993;132(26):653–6. doi: 10.1136/vr.132.26.653 8362471

[38] Nimpaye H, Njiokou F, Njine T, Njitchouang GR, Cuny G, Herder S, et al. *Trypanosoma vivax*, *T*. *congolense* "forest type" and *T*. *simiae*: prevalence in domestic animals of sleeping sickness foci of Cameroon. Parasite. 2011;18(2):171–9. doi: 10.1051/parasite/2011182171 21678793 PMC3671417

[39] McDermott J, Woitag T, Sidibe I, Bauer B, Diarra B, Ouedraogo D, et al. Field studies of drug-resistant cattle trypanosomes in Kenedougou Province, Burkina Faso. Acta Trop. 2003;86(1):93–103. doi: 10.1016/s0001-706x(03)00019-6 12711108

[40] Tchamdja E, Kulo AE, Vitouley HS, Batawui K, Bankole AA, Adomefa K, et al. Cattle breeding, trypanosomosis prevalence and drug resistance in Northern Togo. Vet Parasitol. 2017;236:86–92. doi: 10.1016/j.vetpar.2017.02.008 28288771

[41] Orenge CO, Munga L, Kimwele CN, Kemp S, Korol A, Gibson JP, et al. Trypanotolerance in N’Dama x Boran crosses under natural trypanosome challenge: effect of test-year environment, gender, and breed composition. BMC Genet. 2012;13:87. doi: 10.1186/1471-2156-13-87 23075408 PMC3519672

