# Supplementary material for: A longitudinal two-year survey of the prevalence of trypanosomes in domestic cattle in Ghana by massively parallel sequencing of barcoded amplicons
Source: PLoS Negl Trop Dis. 2022 Apr 20;16(4):e0010300. doi: 10.1371/journal.pntd.0010300 (PMC9060370; doi:10.1371/journal.pntd.0010300)
Supplement: S1 Text — (A) Shows the sequences. (B) Shows the location of the oligos in the relevant parts of the genomes from T. brucei, T. congolense, T. vivax and T. theileri. (DOCX) [file pntd.0010300.s001.docx]

**S1 Text**

**Oligonucleotide sequences for the nested tubulin intergenic PCR**

A. Shows the sequences.

B. Shows the location of the oligos in the relevant parts of the genomes from *T. brucei, T. congolense, T. vivax* and *T. theileri*.

A.

outer forward GGTGAGTTCTCCGAGGCCCGTG

outer reverse CCGTGCTCGTCGCTGATCAC

equimolar mixture CCGTGCTCATCGCTGATCAC

CCGTGCTCGTCACTGATCAC

|  |  |  |  |
| --- | --- | --- | --- |

inner forward GAGGAGGATGTGGGAGGAGTACTA

equimolar mixture GAGGAGGACGTTGGAGGAGTACTA

GAGGAGGACGTGGGAGGAGTACTA

inner reverse CTGATCACYTCCCAGAACTT

B.

*T. brucei*

Nested PCR product 424 bp

GGTGAGTTCTCCGAGGCCCGTGAAGACCTTGCAGCACTTGAGAAGGACTACGAAGAGGTTGGTGCCGAGTCCGCGGATATGGACGGTgaggaggatgtggaggagtactagaaagtgtgacaacgtcgcaccatgtgtaggttttcatttacgttctttctttcttttgtgaatttgttttctgtctcaaatgtttttaattcgcttgggacctatgtttttcttgtttttttgctcaccctttgtgtaggaggcaccctgtcacgtctgtggttgcgtgtatgccttccttccccttattcgcttcttcctgtcgtgtcacacctctttctctctctccctttccgccttttctttcaatcttgttttctcgaccagccctactagaggagaaagaatagtaaccctttcatcaaagaaaatagttcaaacgaattatgcgcgaaatcgtctgcgttcaggctggccaatgcggtaaccagatcggctcaaagttctgggaggtgatcagtgacgagcacgG

*T. congolense*

Nested PCR product 456 bp

GGTGAGTTCTCCGAGGCCCGTGAAGATCTCGCTGCCCTCGAGAAGGATTACGAGGAGGTCGGTGCGGAGTCTGCGGACATGGACGGCgaggaggacgttgaggagtactagagaacaggaaaagaagtgtgtctgtgtggaggatgtagcagtctaatttatatgtatgaccttgttagcatgtttgttgtgaggcttgtgctgatacctggagctctaataggacacctgtgtatgcacgttcggcacaccctcctcagacttttttgtttttgtttcttttttctttatttatcttgttttgtttcctctcattaatgaagtgttccctcttcctgtcccactccgcctgcctggttatcgccgcctacatggcgttccctcaccaccaccaccaccaccctccctccctcctttcgctgcgctgaaagattataatcgtttcaaggaagactaatccaattccatcatgcgtgagatcgtttgtattcaggctggccagtgcggcaaccagattggctccaagttctgggaggtgatcagCGATGAGCACGG

*T. vivax*

Nested PCR product 586 bp

GGTGAGTTCTCCGAGGCCCGTGAGGACCTTGCTGCGCTGGAGAAGGACTACGAGGAGGTTGGCGCGGAGTCCGCGGACATGGAGGGCgaggaggacgtggaggagtactagacgccccgttgttgcgggcctcgcgtgcgcgtatgtatgtatgtgtgtatgtgtgagcggtggtcctcgcgcacgcgcgctcccccaccactctttCttttttcttttttctttttcttttttatgtctgttttgtgttttccttcctttccccctTgggccggtttcttttcccattttttaaatactctttctcctgtttgttccacacttcggTtcatgactcccgccttcccgcgcgtcccctggcggacagcagcggcatgcgcgtgtgcaCagccacgtgtgtgacattgcctcatttgccctgcgcgccccacacccccctccaatttcCcaccgttcccttctcttccctcctttccttgtcccccgtcccgtcccgtcccgccgcacCtgcgcccgtgtctgcaaacgcggccccagaaaagcaagtgcattcacaaagcatccattGagatctaaccaccaagaaaacccaagcgaatatgcgtgagattgtttgtgttcaggcggGccagtgcggcaaccagatcggctccaagttctgggaagtgatcagCGACGAGCACGG

*T. theileri*

Nested PCR product 646 bp

GGTGAGTTCTCCGAGGCCCGTGAGGATCTTGCCGCACTCGAGAAGGACTACGAGGAGGTTGGCGCAGAGTCCGCCGATATGGAGGGCGAGGAGGACGTGGAGGAGTACtagatatgtagagctaccccaggttttctccctatttttcttttttccgcgggatgggcgggttagggagctgtgtgcgcatgttcgtgatgtggtagagagaaagcacactgctgtatggggagggaaaggggaactggcgttgtagcaactgcaactggaggagtgtggtgatgggtggtacacatgtatcaggcgctgacgccccttggcctcatttcatttttctcatttcttgttccactctagctggtctgttgtttcccatctcgctatgtgcttcttttcccattttttttttcttttgttgaccatcgttcaccgtgcgggtacatatgactgtctctctgtttttttttttttctctcttttttttttcttctttttctatttctttctctcccctttgtttgtgtttgctttgaccgctcatgtgtgtcgtgctgtcatcgcatgcgccataactataccgtgaaagaaaaaaacagaatagaagaacctcaagaagagattcaatcgaacaacaattactgaataacagttgaagagaaATGCGCGAGATTGtgtgtgtTCAGGCCGGCCAGTGCGGTAACCAGATCGGTTCAAAGTTCTGGGAGGTGATCAGCGACGAGCACGG
