# Supplementary figures and images for: A longitudinal two-year survey of the prevalence of trypanosomes in domestic cattle in Ghana by massively parallel sequencing of barcoded amplicons
Source: PLoS Negl Trop Dis. 2022 Apr 20;16(4):e0010300. doi: 10.1371/journal.pntd.0010300 (PMC9060370; doi:10.1371/journal.pntd.0010300)

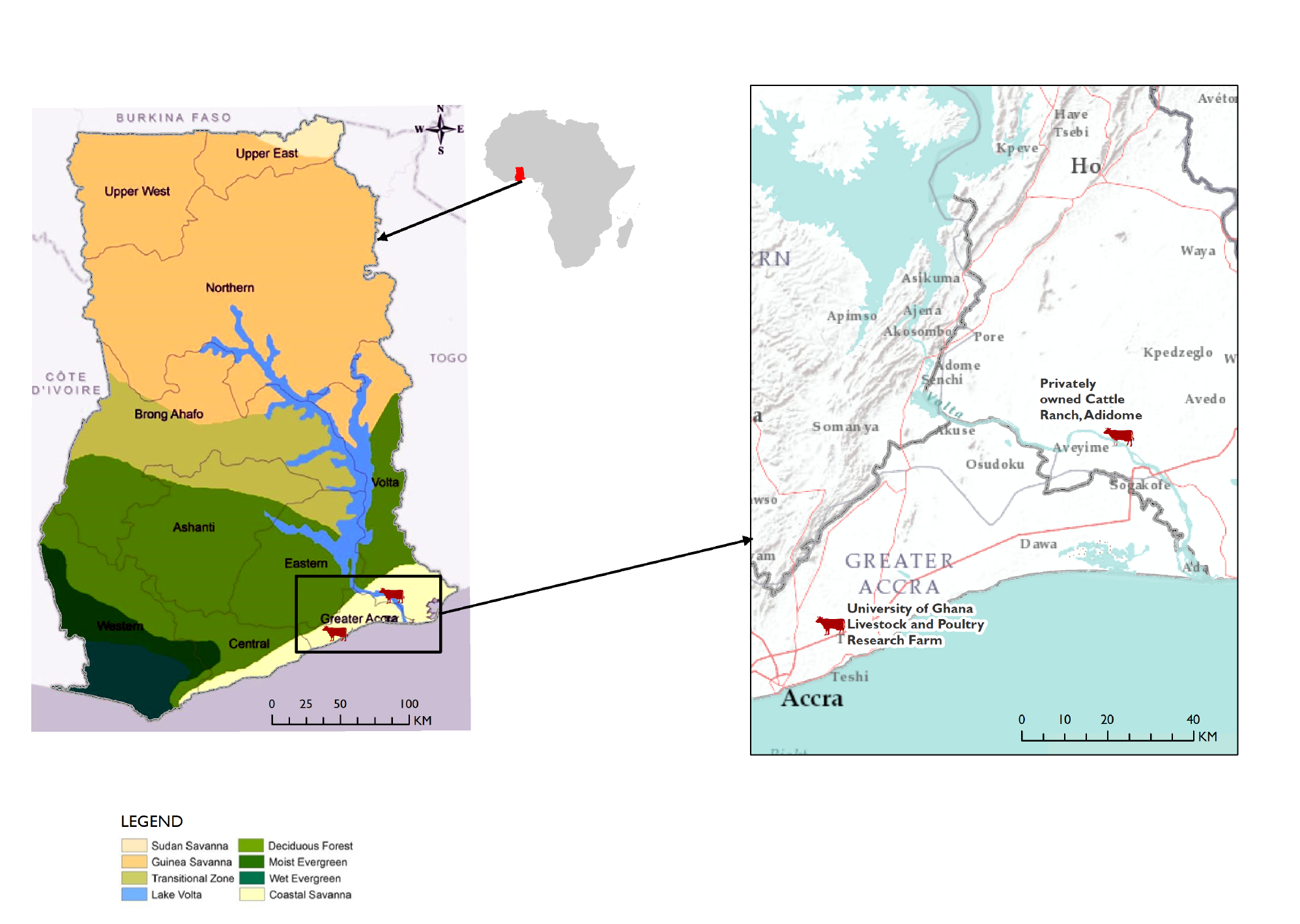

Supplement: S1 Fig — Map was drawn using the ArcGiS version 10. Source map: https://www.arcgis.com/apps/mapviewer/index.html?webmap=a52ab98763904006aa382d90e906fdd5. (TIF) [file pntd.0010300.s002.tif]
